# Sigmoid Lithobezoar associated with iron deficiency anaemia: a rare case report

**DOI:** 10.1093/omcr/omaf252

**Published:** 2025-11-26

**Authors:** Bodhisatya Das, Mahaprasad Pal, Kaustav Nayek, Wasim Akram, Dattatreya Mukherjee, Sumitaksha Banerjee, Aymar Akilimali

**Affiliations:** Department of Pediatrics, Burdwan Medical College and Hospital, Baburbag, Purba Burdwan, West Bengal, 713104, India; Department of Pediatrics, Burdwan Medical College and Hospital, Baburbag, Purba Burdwan, West Bengal, 713104, India; Department of Pediatrics, Burdwan Medical College and Hospital, Baburbag, Purba Burdwan, West Bengal, 713104, India; Department of Pediatrics, Burdwan Medical College and Hospital, Baburbag, Purba Burdwan, West Bengal, 713104, India; Department of Pediatrics, Raiganj Government Medical College and Hospital, Raiganj, Uttar Dinajpur, West Bengal, 733134, India; Department of Pediatrics, Burdwan Medical College and Hospital, Baburbag, Purba Burdwan, West Bengal, 713104, India; Department of Research, Medical Research Circle, Kyeshero, Lusaka rue 218, Postal Code 73 Gisenyi, Goma, North Kivu, DR Congo

**Keywords:** Lithobezoar, iron-deficiency anaemia, pica, obstructed defecation

## Abstract

In this case study, we describe the presentation of a 6-year-old male patient afflicted with iron deficiency anaemia (IDA) who manifested gastrointestinal obstruction attributable to the habitual ingestion of brick dust and soil, a characteristic behaviour associated with pica. The obstructive material, resembling bricks in consistency, necessitated manual extraction from the patient's anus. Subsequently, the patient received oral iron supplementation as part of the therapeutic regimen. While the causative relationship between pica and IDA remains subject to ongoing debate within the scientific community, our findings underscore the importance of implementing routine screening protocols for iron deficiency in the evaluation of lithobezoar cases among paediatric populations.

## Introduction

A bezoar refers to an accumulation of non-digestible foreign material within the gastrointestinal tract. Its occurrence is rare, with a prevalence of less than 1% in the general population [1]. The stomach is where they are typically found, with the intestines being the second most common location. Based on their contents, four different types of bezoars have been identified: Phytobezoar pertains to undigestible plant matter, trichobezoar involves hair, lactobezoar concerns undigested milk in infants, and lithobezoar involves soil and stones [2]. The most common type is called phytobezoar. The most common sources of phytobezoar are foods high in cellulose, such as figs, grapes, and pumpkins [3, 4]. The most common symptom is abdominal pain, and a bezoar can result in potentially fatal consequences like perforation or intestinal blockage. Surgical intervention is typically employed for large or intricate bezoars, while endoscopic or pharmaceutical approaches suffice for managing smaller ones [5]. Prolonged consumption of non-nutritive materials or substances exceeding one month is clinically termed as pica [6].

Iron deficiency continues to be a prominent contributor to anaemia, affecting approximately one-third of the global population. Although prevalent worldwide, iron-deficiency anaemia (IDA), is particularly prevalent among children residing in developing nations [7, 8]. While anaemia represents the severe end of the spectrum of iron deficiency, it has also been linked to diverse neurological symptoms, including cognitive deficits and aberrant behaviours such as pica [6]. The direction of the causal relationship between iron deficiency and pica remains unclear despite being established. While some studies have shown that pica resolves when IDA treatment is received, others have reported that cases remain unresolved even after iron therapy [9, 10]. Numerous studies have established associations between pica and various conditions and factors, including deficiencies in essential minerals and vitamins such as iron, and less commonly, zinc, calcium, thiamine, niacin, and vitamin C [11, 12]. Additionally, pica has been correlated with several illnesses, including depression, obsessive-compulsive disorder (OCD), pathological anxiety, schizophrenia, emotional disturbances, and developmental disabilities such as autism spectrum disorders and severe/profound intellectual disability [13–15].

Thus, here we present a case report of a 6-year-old boy with iron-deficiency anaemia, who presented with features of pica which eventually revealed lithobezoar.

## Case report

A 6-year-old boy from Bankura district of West Bengal was brought to the department of Paediatrics with a complaint of not passing stool since last 2 days. The patient presented with absolute constipation at the time of admission with history of pain abdomen. He belongs to a family of lower Socioeconomic status. His anthropometric values are, weight: 15.9 kg, height: 112.0 cm, Body Mass Index (BMI): 12.7 kg/m^2^. Mid-Upper Arm Circumference (MUAC): 13.4 cm, Head Circumference (HC): 49.9 cm, Chest Circumference (CC): 53.8 cm. The child’s height of 112.0 cm at 6 years is above the 3rd percentile, indicating acceptable linear growth. His weight of 15.9 kg for this height is also above the 3rd percentile, but still on the lower side. BMI is 12.7 kg/m^2^, which falls below the 3rd percentile, indicating thinness. MUAC is 13.4 cm, also below the 3rd percentile, supporting a diagnosis of undernutrition. Overall, the child is malnourished, with normal height but evidence of acute undernutrition based on BMI and MUAC. His mother gives a history that he often used to eat inconsumable substances such as coal dust and soil from surrounding and recently, he consumed brick dust while playing with his friends.

**Figure 1:**
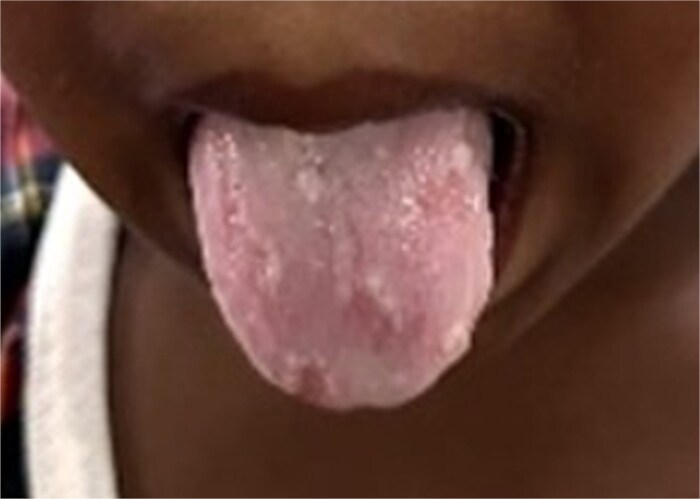
Pallor in the tongue.

**Figure 2:**
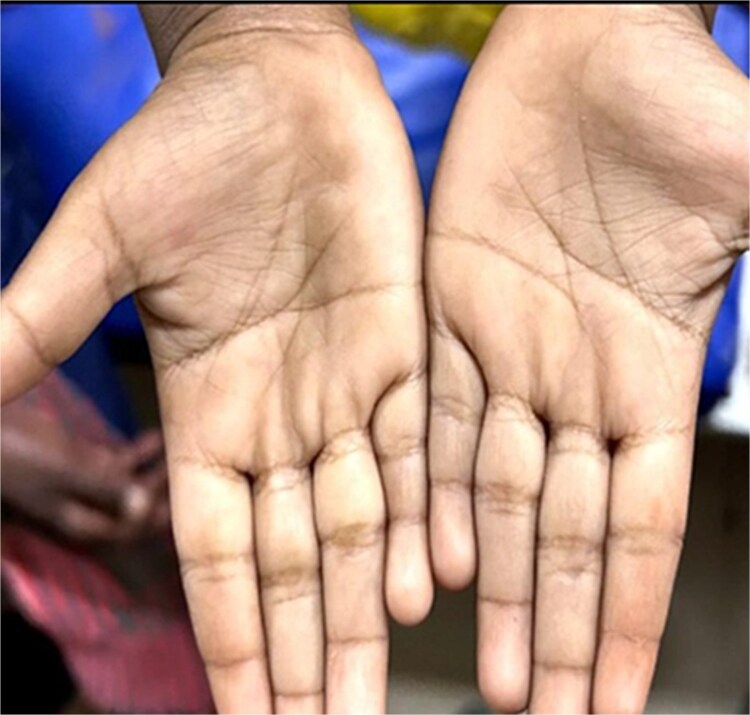
Pallor in the hand.

**Figure 3:**
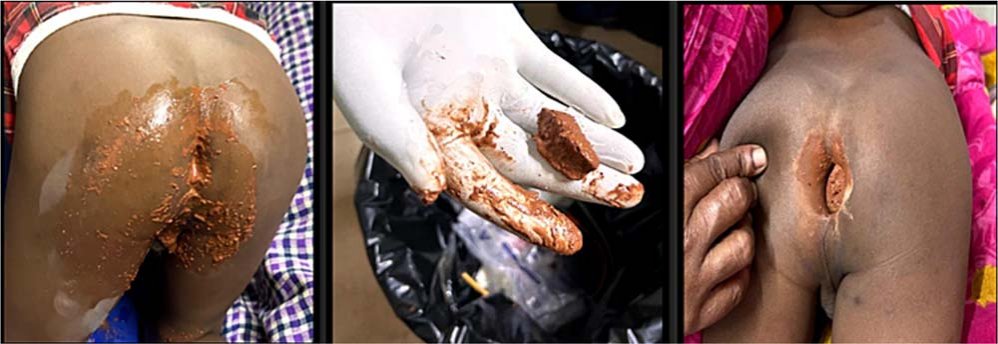
Examination of anus revealed a hard substance with brick-like appearance obstructing his rectum.

Clinical examination showed severe pallor in his tongue, lower eyelid, palms and soles. (Figs 1 and 2) On physical examination the abdomen was distended, on percussion hyper resonant note was present and bowel sound was increased on auscultation. Examination of anus revealed a hard substance with brick-like appearance obstructing his rectum. (Fig. 3) After admitting the patient, ultrasonography of whole abdomen and a digital straight X-ray abdomen were done as initial investigations. No significant radiological findings were obtained. During admission, laboratory report showed haemoglobin of 2.0 gm%, serum iron 8 μg/dl, total iron binding capacity 989 μg/dl, serum transferrin 2.13% and serum ferritin 8.2 ng/dl. This was a clear indication of severe iron deficiency anaemia. The CBC report during the admission is mentioned in Table 1. He received one unit of PRBC before operation.

**Table 1:** CBC report during admission.

Hb	2.0 gm%
WBC	13 000/microlit.
Platelet	70 000/microlit.

He was shifted to operation theatre for further management. Emergency laparotomy was done to extract the lithobezoar. The brick-like materials from lower part of rectum were removed manually. After that he was shifted to Paediatric intensive care unit. Patient received two units of PRBC after Operation. After transfusion, haemoglobin was 5 grams%.

The patient was admitted in the hospital for 14 days. The value of haemoglobin during discharge was 6.7 gram%. During discharge, oral iron therapy was prescribed (5 mg/kg/day) to him and his parents were advised for regular check-up. On the follow up after 7 days, he reported no further obstruction in defecation although the pica was not completely resolved. Psychiatric evaluation found no major psychological problems and his parents were informed that his pica will gradually disappear with age and improvement in iron profile.

## Discussion

The symptoms of bezoars can vary based on the amount, kind, and location of undigested foreign material present. However, common symptoms include halitosis, nausea, vomiting, anaemia, abdominal pain, distention, and bleeding in the upper gastrointestinal tract [16]. According to Iwamura et al. [17], the most typical symptom was abdominal pain, which was followed by tarry or bloody stools. An ongoing irritation of the stomach mucosa may infrequently result in symptoms of pancreatitis, peritonitis, ulceration, and perforation [18, 19]. According to Lee et al. [20], the most frequent side effect of bezoars (41.2%) was gastric ulceration. In contrast, patient of the present case showed only obstruction in defecation with no complaint of abdominal pain.

The primary diagnostic instrument is an extensive clinical history. Endoscopy stands out as the optimal choice for minimally invasive treatment of gastric bezoars, while both CT scanning and endoscopy serve as confirmatory diagnostic modalities [21]. Nonetheless, in cases where bezoars are situated in the small intestine, endoscopy alone may not suffice for accurate diagnosis, warranting the use of CT imaging. Furthermore, CT imaging enables visualization of multiple small bowel bezoars, proving advantageous for surgical planning in affected patients [22]. In the stomach's fundus, phytobezoars and lithobezoars are usually discovered as a single mass, though they can also appear as several masses. Trichobezoars most frequently occur in the stomach, but they can also occur in the small intestine. A bezoar's colour varies according to its composition. The colour of a phytobezoar can be black, yellow-green, or beige. A lactobezoar is white because of the mucus and milk content [23]. Although no radiological findings were present in this case, anal examination and visual appearance of the material confirmed it to be brick.

Surgical, endoscopic, and pharmaceutical methods are the main approaches to treatment [24]. Small stomach bezoars can be treated non-surgically and conservatively. Apart from mechanical methods, non-surgical techniques such as gastric lavage or endoscopic fragmentation employing various tools including polypectomy snares, biopsy forceps, alligator forceps, baskets, lithotripters, argon plasma coagulation, needle-knives, or extracorporeal lithotripsy have been utilized. Additionally, substances such as acetylcysteine, cellulase, papain, and cola have also been employed in the management of bezoars [21, 25]. Kurt et al. [26] reported on a novel bezoar fragmentation snare device known as a bezoaratom in a 2014 case report. A Chinese study that used an endoscope and a mini-explosive laser technique discovered that 260 patients had a nearly 100% cure rate [27]. When endoscopic and medication interventions fail to resolve the issue, or if the bezoar is substantial, or complications such as perforation, penetration, or obstruction arise, surgical intervention becomes necessary. Surgery is typically performed under general anaesthesia, and the choice between open or laparoscopic approaches depends on the specific circumstances and the surgeon's expertise. When bezoars are found in the stomach, a gastrostomy procedure is typically used; however, an enterotomy might be suitable for bezoars found in the small bowel. In rare cases, bezoars can be found in the small intestine as well as the stomach, requiring both procedures. As per a review published by Masaya Iwamuro et al, they have mentioned that in less than 0.5% patients undergoing esophagogastroduodenoscopy examinations have bezoar in stomach 0.4%–4.8% patients presented with intestinal obstruction have bezoar in the small intestine. [17] In this instance, the large size and lack of prior experience with laparoscopic surgery with bezoars led to the decision to remove the bezoar by open surgery rather than endoscopic fragmentation [28]. Our patient did not require any medicinal or surgical treatment as the obstructing material could be removed manually.

Remarkably, pica behaviour tends to occur when patients are least supervised, and individuals often keep their pica habits secretive, hesitating to disclose them. Consequently, pica symptoms may remain undetected unless healthcare providers specifically inquire about them. Notably, pica appears to be closely linked with iron deficiency anaemia, with many cases demonstrating cessation of abnormal eating and chewing behaviours following iron supplementation. Some theories suggest that individuals with anaemia may chew ice for its perceived analgesic effects, potentially alleviating glossal pain associated with iron deficiency. Melissa G Hunt et al. has written a wonderful paper on this, relationship between pagophagia (compulsive ice chewing) and iron deficiency anaemia. [29] However, it's worth noting that substances like rubber bands and foam lack known analgesic properties [30, 31]. In this case, the patient was diagnosed with severe iron deficiency anaemia and prescribed with oral iron supplements.

## Conclusion

Although the pathogenesis of pica remains inadequately understood, its associated risk factors are well-documented. Given that the identification of pica is frequently overlooked, maintaining a high index of suspicion is crucial, particularly when individuals with known risk factors exhibit suggestive signs and symptoms. Pica can serve as a significant indicator of iron deficiency, particularly in paediatric populations. Consequently, clinicians should be prompted to investigate potential underlying causes of unusual substance cravings, such as IDA due to nutritional deficiencies or chronic blood losses.

## Summary

Lithobezoar Associated with Iron Deficiency Anaemia in a 6 years old boy came from a low socioeconomic family at West Bengal, India.
